# Grip strength as a predictor of depressive symptoms among vulnerable elderly Europeans with musculoskeletal conditions

**DOI:** 10.1038/s41598-021-00751-3

**Published:** 2021-10-29

**Authors:** Priscila Marconcin, Adilson Marques, Duarte Henriques-Neto, Élvio R. Gouveia, Gerson Ferrari, Miguel Peralta, Andreas Ihle

**Affiliations:** 1 Faculdade de Motricidade Humana, Universidade de Lisboa, Lisbon, Portugal; 2 CIPER, Faculdade de Motricidade Humana, Universidade de Lisboa, Lisbon, Portugal; 3 ISAMB, Universidade de Lisboa, Lisbon, Portugal; 4 Department of Physical Education and Sport, University of Madeira, Funchal, Portugal; 5 LARSYS, Interactive Technologies Institute, Funchal, Portugal; 6 Laboratorio de Ciencias de la Actividad Física, el Deporte y la Salud, Facultad de Ciencias Medicas, Universidad de Santiago de Chile, USACH, Santiago, Chile; 7 Center for the Interdisciplinary Study of Gerontology and Vulnerability, University of Geneva, Geneva, Switzerland; 8 Swiss National Centre of Competence in Research LIVES-Overcoming Vulnerability: Life Course Perspectives, Lausanne, Switzerland; 9 Department of Psychology, University of Geneva, Geneva, Switzerland

**Keywords:** Diseases, Health care

## Abstract

The present study aimed to investigate the grip strength (GS) discrimination capacity and cutoffs points for depressive symptoms among vulnerable elderly individuals with musculoskeletal conditions. The Survey of Health, Aging, and Retirement in Europe wave 6 was analyzed. GS was measured by a handgrip dynamometer, and EURO-D scale was used to assess depressive symptoms. GS cutoff values for depressive symptoms were calculated using the receiver operating characteristics curve. 2206 participants, mean age 74.0 (73.7–74.3), 78.8% with osteoarthritis/other rheumatism, enrolled in the study. Sensitivity varies between 0.44 (men, ≥ 85 years) and 0.82 (men, 75–84 years), and specificity varying between 0.35 (women, 75–84 years) and 0.70 (men 75–84 years). GS is associated with depressive symptoms just for women and it is not possible to use a GS cutoff point for screening depressive symptoms for vulnerable men and women with musculoskeletal conditions over the age of 65 years.

## Introduction

The prevalence of musculoskeletal (MSK) conditions is high worldwide^1,2^, mainly among elderly individuals, who also have age-related changes in muscle, bone, fat, and the nervous system^3^. MSK conditions are the most common cause of pain and disability^2^, and are associated with daily fatigue and a consequent decrease in physical activity levels^4^.

Weakness, evaluated by grip strength (GS), is a vulnerability feature to identify sarcopenia^5^, and frailty syndrome^6^. GS is a simple and inexpensive proxy of overall muscle strength; it is often used for clinical and epidemiological studies^7^. It is a reliable measure to predict undernutrition risk^8^, metabolic syndrome^9^, mobility limitation^10^, and mental health^11,12^.

MSK conditions are likely to be chronic, present pain as the main symptom, and might have a psychological impact^13,14^. The prevalence of pain in depressed cohorts, and depression in pain cohorts, is higher than when these conditions are individually examined^15^. Although depression is present in MSK patients^16^, the diagnosis is frequently missed in primary care settings^17^, largely because patients deny mental illness^18^. Depression and pain share biological pathways and neurotransmitters, which have implications for the treatment of both concurrently^19,20^. Therefore, individuals with MSK conditions must be screened for depressive symptoms.

The association of GS and depressive symptoms is well studied in different samples^11,12^. But, a current study shows that this association depends on chronic disease, being different in individuals with arthritis disease^21^. For individuals with MSK conditions, who have pain and find it difficult to stay seated to complete questionnaires, a GS cutoff point that helps to screen depressive symptoms could be an excellent approach. In this study, we intended to explore the relationships among muscle weakness and depressive symptoms in vulnerable elderly individuals with MSK conditions (e.g. osteoarthritis/other rheumatism and rheumatoid arthritis). Therefore, this study aimed to examine GS discrimination capacity for depressive symptoms and to determine the GS cutoff point to identify depressive symptoms, by gender and age group, among elderly individuals with MSK conditions (e.g. osteoarthritis/other rheumatism and rheumatoid arthritis).

## Methods

### Study design

This study used data from the population-based Survey of Health, Aging, and Retirement in Europe (SHARE) wave 6 (2015). SHARE is a multidisciplinary and cross-national panel database of microdata on health, socio-economic status, and social and family networks among individuals aged 50 or older. SHARE covers 27 European countries and Israel. More details about the project can be found elsewhere^22^ and on the website (http://www.share-project.org/home0.html). The SHARE protocol was approved by the Ethics Committee of the University of Mannheim, and by the Ethics Council of the Max-Planck-Society for the Advancement of Science, verifying the procedures to guarantee confidentiality and data privacy. Written informed consent was obtained from all participants involved in the study. All procedures were performed in accordance with ethical guidelines and regulations in accordance with the Declaration of Helsinki^23^.

### Participants

The sample of SHARE wave 6 included 68,231 participants. In this study, the population included those who reported being clinically diagnosed with MSK conditions (e.g. osteoarthritis/other rheumatism and rheumatoid arthritis) and were older than 65 years old. Moreover, all participants must have reported depressive symptoms, completed the GS assessment, and reported information that allowed for their characterization (gender, age, education level, weight, height). The final sample consisted of 2206 participants (733 men and 1473 women), mean age 74.04 (SD = 7.12), 690 with rheumatoid arthritis, and 1738 with osteoarthritis/other rheumatism. Participants were from 18 countries (Austria, Germany, Sweden, Spain, Italy, France, Denmark, Greece, Switzerland, Belgium, Israel, Czech Republic, Poland, Luxembourg, Portugal, Slovenia, Estonia, and Croatia).

Face-to-face interviews to answer the questionnaires, lasting approximately 90 min, at the participant’s home, were used to collect data. The questionnaires were translated by translation experts according to the country. The first draft SHARE questionnaire was piloted with the help of the National Centre for Social Research.

### Measures

The GS was assessed twice on each hand using a handgrip dynamometer (Smedley, S Dynamometer, TTM, Tokyo, Japan, 100 kg). The grip strength test was explained and demonstrated before the assessment, and participants had the opportunity to practice. Participants could sit or stand, with their elbow at a 90° angle, the wrist in a neutral position (while keeping the upper arm tight against the trunk), and the inner lever of the dynamometer adjusted to the hand. Participants squeezed the dynamometer with their hand, as hard as possible, for 5 s. Values were recorded twice for each hand, alternating between left and right hands. Valid measurements were values of two measurements in one hand that differed by less than 20 kg^24^. All GS measurements with values of 0 kg or ≥ 100 kg were excluded; measurements with only GS recorded on one hand were also excluded.

Depressive symptoms were measured by the EURO-D 12-item scale, covering depression, pessimism, suicidality, guilt, sleep, interest, irritability, appetite, fatigue, concentration, enjoyment, and tearfulness (in the last month). The scale details, and their validation, are described elsewhere^25^. It is done by summing item scores for individual symptoms, that are coded as 0 and 1, when they are ‘not present’ and ‘present’ respectively. A total score ranges from 0 to 12, with a higher score indicating more depressive symptoms. Depressive symptoms was analyzed as a continuous variable and a dichotomic variable. For this study, a cutoff point of ≥ 4 points would diagnose clinically significant depressive symptoms^25^.

Based on a list of 16 diseases, participants were asked to report the presence or absence of each disease by disclosing whether they were previously diagnosed by a doctor. For this study, participants who received the clinical diagnosis of rheumatoid arthritis and/or osteoarthritis/other rheumatism were included.

Potential confounders included gender, age, education level, body mass index (BMI), physical activity and country. Age was organised into three groups: 65–74, 75–84, and ≥ 85 years. Education level was grouped as low, middle, or high, according to the International Standard Classification of Education 1997 (ISCED-97). The BMI was calculated from self-reported height and weight, dividing the weight (kg) by the square of height (m).

### Statistical analysis

Descriptive statistics [including mean, percentages, and 95% confidence interval (CI)] for all variables were calculated for the total sample, and by sex. The GS discrimination capacity for depressive symptoms reflects the capability of the model to distinguish between participants, with and without depressive symptoms, using GS values. The predictive capability of GS alone was analyzed using the area under the curve (AUC), which ranges between 0.5 and 1.0 for sensible models^26^. The receiver operating characteristics (ROC) curves that created an AUC above 0.50, for the GS, were considered to have sufficient discrimination ability between participants with and without depressive symptoms (95% CI were performed using the nonparametric approach)^27^. The GS cutoff values for depressive symptoms were calculated using the ROC curve coordinates with Youden’s index^28^. The cutoff values represent the best trade-off between sensitivity and specificity values for GS in each sex age-group. Since GS varies significantly with age and sex, the analysis was stratified for both sex and age group. Logistic regression models were used to quantify the odds of having depressive symptoms below or above the GS cutoff value. All models were adjusted for age, education level, and BMI; stratified for sex and age group. Data analysis was performed using IBM SPSS Statistics v.22 (SPSS Inc., an IBM Company, Chicago, Illinois, USA).

## Results

Participants’ characteristics are presented in Table 1. In total, 2206 individuals (1473 women, mean age 74, 95% CI 73.7–74.3) were analyzed. Osteoarthritis/other rheumatism was the most prevalent MSK condition (78.8%, 95% CI 77.1–80.5). More women had depressive symptoms than men (33.5%, 95% CI 31.1–35.9). The mean GS for men and women was 38.6 (95% CI 37.9–39.3) and 23.5 (95% CI 23.2–23.8), respectively.

**Table 1:** Participant’s characteristics,

	% or M (95% CI)		
	Total (n = 2206)	Men (n = 733)	Women (n = 1473)
Age	74.0 (73.7–74.3)	74.4 (73.8–74.9)	73.9 (73.5–74.2)
**MSK conditions**			
Rheumatoid arthritis (RA)	31.3 (29.3–33.2)	32.2 (28.8–35.6)	30.8 (28.5–33.2)
Osteoarthritis (OA)	78.8 (77.1–80.5)	76.5 (73.5–79.6)	79.8 (77.9–82.0)
Both OA/RA	10.1 (8.8–11.3)	8.7 (6.7–10.8)	10.7 (9.1–12.3)
**Chronic diseases**			
Less than 2	58.8 (56.8–60.9)	58.9 (55.4–62.5)	58.8 (56.3–61.3)
2 or more	41.2 (39.1–43.2)	37.5 (37.5–44.6)	41.2 (38.7–43.7)
**Education level**			
Low	44.1 (42.0–46.2)	39.3 (35.7–42.9)	46.4 (43.9–49.0)
Medium	35.3 (33.3–37.3)	36.0 (32.5–39.5)	35.0 (32.5.0–37.4)
High	20.6 (18.9–22.3)	24.7 (21.6–27.9)	18.6 (16.6–20.6)
BMI	26.5 (26.3–26.6)	26.8 (26.5–27.1)	26.3 (26.1–26.5)
Grip strength (kg)	28.5 (28.1–28.9)	38.6 (37.9–39.3)	23.5 (23.2–23.8)
EURO-D	2.6 (2.5–2.6)	2.1 (1.9–2.2)	2.8 (2.7–2.9)
**Depression**			
No	70.1 (68.2–72.0)	77.4 (74.3–80.4)	66.5 (64.1–68.9)
Yes	29.9 (28.0–31.8)	22.6 (19.6–25.7)	33.5 (31.1–35.9)

*M* mean, *CI* confidence interval, *BMI* body mass index.

The discriminant capacity of GS for depressive symptoms including AUC, sensitivity, specificity, and cut-off values, stratified by sex and age group, is presented in Table 2. AUC was above 0.5 for both sexes and age groups, indicating a discriminant capacity. Furthermore, sensitivity varied between 0.84 for men between 75 and 84 years and 0.44, for men ≥ 85 years of age. Specificity varied between 0.17, for men ≥ 85 years, and 0.60 for women ≥ 85 years. Overall, sensitivity values were always higher than specificity values within each group. GS cut-off values for discriminating the presence of depressive were the following: men aged 65–74, 41.5 kg; men 75–84, 40.5 kg; men older than 85 years, 23.5 kg; women aged 65–74, 24.5 kg; women 75–84, 20.5 kg; women older than 85 years, 19.5 kg.

**Table 2:** Receiver operating characteristic for handgrip strength as an indicator of not having depression.

	Men (733)			Women (1473)		
	65–74 years	75–84 years	≥ 85 years	65–74 years	75–84 years	≥ 85 years
**Without depression**						
n	332	173	62	609	282	89
Grip strength (kg)	42.8 (41.8–43.7)	35.6 (34.3–36.8)	29.7 (27.9–31.5)	25.9 (25.5–26.4)	22.8 (22.2–23.5)	17.9 (16.8–19.0)
**With depression**						
n	86	62	18	272	161	60
Grip strength (kg)	40.8 (39.0–42.7)	33.8 (32.0–35.6)	27.6 (22.5–32.6)	23.9 (23.1–24.8)	20.9 (20.0–21.8)	15.9 (14.7–17.1)
AUC (95% CI)	0.55 (0.48–0.62)	0.57 (0.49–0.65)	0.59 (0.42–0.75)	0.58 (0.54–0.62)	0.59 (0.53–0.64)	0.61 (0.52–0.70)
Grip strength cutoff for depression (kg)	41.50	40.50	23.50	24.50	20.50	19.50
Sensitivity	0.50	0.84	0.44	0.53	0.46	0.82
Specificity	0.39	0.70	0.17	0.40	0.35	0.60

*AUC* area under the curve.

The odds ratio (OR) for the association between being above the GS cutoff value and the risk of having depressive symptoms, for men and women, (and by age groups) can be found in Table 3. For men, being above the GS cutoff value was not significant in any age range. However, for women, being above the GS cutoff value was significantly associated with lower odds of having depressive symptoms, in all age ranges. Women aged 65 to 74, 75 to 84, and older than 85 years old had a statistically significant lower chance of presenting with depressive symptoms (34%, 39%, and 47%, respectively).

**Table 3:** The odds ratio for having depression symptoms according to being below or above the GS cutoff value for men and women among age groups.

	Model adjusted^1^
**Men**	
65–74 years old	
Below GS	1 (Ref)
Above GS	0.82 (0.55–1.22)
75–84 years old	
Below GS	1 (Ref)
Above GS	0.84 (0.57–1.24)
≥ 85 years old	
Below GS	1 (Ref)
Above GS	1.71 (0.36–1.39)
**Women**	
65–74 years old	
Below GS	1 (Ref)
Above GS	0.72 (0.57–0.92)
75–84 years old	
Below GS	1 (Ref)
Above GS	0.68 (0.53–0.87)
≥ 85 years old	
Below GS	1 (Ref)
Above GS	0.58 (0.45–0.76)

^1^Analysis were adjusted to sex, age, education level, chronic disease, BMI, physical activity and country.

*GS* grip strength.

## Discussion

The present study aimed to explore the grip strength (GS) discrimination capacity for depressive symptoms, and to determine the best GS cutoff value to identify depressive symptoms by sex and age group among vulnerable elderly individuals with musculoskeletal (MSK) conditions (e.g. osteoarthritis/other rheumatism and rheumatoid arthritis). Data came from a transnational population-based study comprising 18 European countries. It was found that the area under the curve (AUC) was just slightly above the acceptable (0.5) for men and women. Also, the sensitivity and specificity values were below the desired, except for men between 75–84 years and women older than 85 years. For men aged 75–84 years, and for women older than 85 years, GS of below 40.5 kg and 19.5 kg, respectively, could be an important discriminant to identify depressive symptoms.

The higher depressive symptoms in women seen this study corroborates previous research^29,30^. Also, the present study observed a significant association between being above the GS cutoff value and low depressive symptoms for women of all age ranges. Being above the GS cutoff value decreases the odds of having depressive symptoms in 28%, 32%, and 42%, aged 65–74, aged 75–84, and older than 85, respectively. This gender difference was also observed in other studies, in which a stronger association between GS and depressive symptoms was observed among females in comparison to males^31,32^. However, the role of gender in this association of GS and depressive symptoms is not yet clear. Important factors related to psychosocial reserve should be included in future research to better understand this sexual dimorphism.

Depressive symptoms were analyzed by EURO-D scale, which is a widely used scale to assess depressive symptoms in late-life^33–35^. We used a cutoff point of ≥ 4 points to diagnose clinically significant depressive symptoms, which has been used in other studies^21,25,36–39^, but this cutoff value did not consider a chronic condition. Comparing our study to another with a similar sample (aged older than 50 years)^21^, the prevalence of depressive symptoms is quite higher in our analyses, 29.9% comparing with 20.7%. The difference is that our sample has MSK conditions included. Studies already show that individuals with MSK conditions have intense depressive symptoms^16,40^. Higher levels of depressive symptoms were associated with a poor self-perception of physical health and being women^34^. Therefore, this cutoff point might not be the most suitable for our sample, that is, depressive symptoms could be underdiagnosed.

In the present study, the overall score in GS achieved is lower compared to other studies with samples that did not present MSK conditions^21,36^. This is expected, as MSK conditions and sarcopenia are involved in the pathway of chronic systemic inflammation. This pro-inflammatory state is characterized by a higher catabolic stimulus in detriment to anabolic metabolism, contributing to muscle atrophy^41^. However, the GS cutoff point of our study was too high. For men, the value was 41.5 kg and 40.5 kg (aged 65–74 and 75–84, respectively), while for screening sarcopenia the cutoff point is 27 kg. For women, the value was 24.5 kg and 20.5 kg (aged 65–74 and 75–84, respectively), while for screening sarcopenia the cutoff point is 16 kg^5^. Another study estimates the value of screening mobility limitation, and for men it was 25.8 kg, while for women it was 17.4 kg^10^.

A study that explored the GS as a predictor of depressive symptom in middle-aged and older adults, with different chronic conditions, founded that GS cut-off values were 43.5 kg for men and 29.5 kg for women aged 50–64 years, 39.5 kg for men and 22.5 kg for women aged ≥ 65 years^37^. We can see that the values comparing the same age are quite similar.

To understand the causal mechanism of the relationship between GS and depressive symptoms, it is essential to analyze this relationship in specific samples, such as patients with MSK. Studies show that this causal mechanism is related to functional capacity. Low GS is not only highly associated with a decrease in functional capacity and physical functioning, it is strongly connected to an increase in depressive symptoms^12^. Therefore, this causal mechanism seems to be different in MSK samples. Depressive symptoms in samples with MSK conditions may be more related to the presence of pain than to a decrease in strength, and functional limitation. The presence of pain negatively affects the recognition and treatment of depression^15^. In samples that present pain as the major complains, as occurs with MSK patients, it is difficult to screen depressive symptoms, because both conditions almost always coexists^42^.

Also, it is important to understand the role of physical activity on the association between grip strength and depressive symptoms. Physical activity levels is associated with increase functional capacity^43^ and high GS^44^. The present study adjusted the analysis for physical activity variable.

Several limitations should be considered. The study design was cross-sectional, exploring the association between variables, but not the cause-effect or direction of the relationships. To assure that all included individuals suffered from musculoskeletal conditions at the same point in time when assessing GS and screening for depressive symptoms, only wave 6 could be used since this is the only wave with this fine-grained information on musculoskeletal conditions and therefore we cannot identify individuals with regard to whether they already had (or had not) these specific health conditions during the prior waves and/or were cured after wave 6. There may also be other variables influencing GS or depressive symptoms (e.g. pain) that were not explored in the current study. Also, GS assessment presents some issues, like no information about participants’ hand size and no consideration about the cultural variation of the use of GS in daily social routines, which must be considered, according to the literature^45^. However, this study used a large and diverse sample of vulnerable elderly individuals from different European countries, covering an extensive range of sociodemographic and clinical characteristics. GS is not a good measure to predict depressive symptoms in this population. However, for women, GS is significant and inversely associated with depressive symptoms. Further prospective studies or randomized trials are necessary to clarify these findings.
